# Impulsivity and the 5-HTTLPR Polymorphism in a Non-Clinical Sample

**DOI:** 10.1371/journal.pone.0016927

**Published:** 2011-02-28

**Authors:** Guilherme M. Lage, Leandro F. Malloy-Diniz, Lorena O. Matos, Marisa A. R. Bastos, Suzana S. C. Abrantes, Humberto Corrêa

**Affiliations:** 1 College of Human, Social and Health Sciences, FUMEC University, Belo Horizonte, Minas Gerais, Brazil; 2 Institute of Biological Sciences, Neuroscience Graduate Program, Universidade Federal de Minas Gerais (UFMG), Belo Horizonte, Minas Gerais, Brazil; 3 Department of Psychology, College of Philosophy and Human Sciences, Universidade Federal de Minas Gerais (UFMG), Belo Horizonte, Minas Gerais, Brazil; 4 Department of Mental Health, College of Medicine, Universidade Federal de Minas Gerais (UFMG), Belo Horizonte, Minas Gerais, Brazil; 5 Sainte Anne Hospital-University Paris Decartes, Paris, Île-de-France, France; University of Michigan, United States of America

## Abstract

**Background:**

Impulsivity has been associated with serotonergic system functions. However, few researchers have investigated the relationship between a polymorphism in the promoter of the serotonin transporter gene (5-HTTLPR) and the different components of impulsivity in a non-clinical population. The aim of this study was to investigate the relationship between a polymorphism in the promoter region of the serotonin transporter gene (5-HTTLPR) and the different components of impulsivity in a non-clinical population.

**Methodology/Principal Findings:**

We administered two neuropsychological tests, the Continuous Performance Task and the Iowa Gambling Task, to 127 healthy participants to measure their levels of motor, attentional and non-planning impulsivity. Then, these participants were grouped by genotype and gender, and their scores on impulsivity measures were compared. There were no significant differences between group scores on attentional, motor and non-planning impulsivity.

**Conclusions/Significance:**

Our results suggest that 5-HTTLPR genotype is not significantly associated with subsets of impulsive behavior in a non-clinical sample when measured by neuropsychological tests. These findings are discussed in terms of the sensitivity of neuropsychological tests to detect impulsivity in a non-clinical population and the role of gender and race in the relationship between the 5-HTTLPR and impulsivity.

## Introduction

Acting without forethought is considered one of the main behavioral expressions of impulsivity, as well as one of the most common definitions found in the literature [1], [2]. Nevertheless, some authors argue that impulsivity manifests in different facets. For instance, Barrat separated impulsive behavior into three components: motor (action without thinking), attentional (lack of focus on the task at hand), and non-planning (orientation towards the present, rather than towards the future) [3], [4]. Bechara's model [5], [6] has many similarities to Barrat's model [3] but associates the three facets of impulsivity with neural correlates.

Bechara [6] argues that motor impulsivity is associated with posterior regions of the orbitofrontal/ventromedial prefrontal cortex, including the basal forebrain. The cognitive impulsivity (analogue to the non-planning impulsivity) is associated with the anterior part of the orbitofrontal/ventromedial prefrontal cortex, including the frontal pole. Bechara [6] also discusses another cognitive type of impulsivity, concerning working memory and the ability to inhibit irrelevant information held in working memory, and to focus on the task at hand. This type of impulsivity is linked to the dorsolateral prefrontal cortex, and it may be analogous to the attentional impulsivity.

Association between impulsivity and biological substrates has been found not only in anatomo-functional features, but also at the molecular level. Impulsivity is in part genetically determined and is somewhat under serotonergic modulation [7], [8]. The serotonin transporter gene is of particular interest because the magnitude and duration of serotonergic activity is regulated mainly by the serotonin transporter protein (5-HTT), which controls the uptake of serotonin from the synaptic cleft [9]. Furthermore, this gene has a functional polymorphism in its regulatory region (5-HTTLPR which regulates the transcription of the 5-HTT. Initially, two variants, a long one (L) and a short one (S) were described [9], with either a 44-bp insertion [long (L)-allele] or deletion [short (S)-allele]. In 2000, Hu et al. [10] described a third functional allele, L_G_, with an A>G polymorphism at position 6 of the first of two 22-bp imperfect repeats that define the 16-repeat L allele. The S and L_G_ alleles are associated with a lower expression of the 5-HTT relative to the L_A_ allele.

Some previous studies have examined the relationship between impulsivity and the 5-HTTLPR in non-clinical populations [11], [12], [13]. However, to our knowledge, only two studies investigated the association between the different facets of impulsivity (motor, attentional and non-planning) and the 5-HTTLPR. In both studies only biallelic analyses were conducted (alleles L and S). Sakado et al. [14] administered the BIS-11 scale, a self-report questionnaire, to 123 subjects to investigate the association among the different components of impulsivity and the 5-HTTLPR.Their results showed that the SS group, compared to the LL and LS groups, scored higher on the overall BIS-11 scale as well as on the attentional subscale. On the other hand, Roiser et al. [7], also using the BIS-11 to study a small sample of 30 subjects, did not find an association between genotypes and impulsivity. However, laboratory behavioral tests are more reliable than self-report questionnaire like BIS-11 because behavioral tests are independent of recall and interpretation of past behavior [1], [12]. Furthermore, laboratory tests can be chosen to assess specific neuropsychological functions.

The continuous performance test (CPT) is a usual laboratory test measuring impulsivity, and it requires the individual to make rapid evaluation/discrimination of presented stimuli to decide whether or not to respond. Traditionally, the index used to assess impulsivity related to inhibition dyscontrol has been the responses to non-target stimuli (called “commission errors”) [15], [16]. On the other hand, attentional impulsivity is assessed by the fails to attend the target stimulus, called “omission errors” [17].

Maintenance of a high risk strategy on the Iowa Gambling Task (IGT) reflects sustained engagement of a particular behavior despite ongoing evidence that it is dysfunctional. IGT models real-life decision-making, specially the type of decisions that are consistent with the construct of cognitive/non-planning impulsivity [4], [17], [18].

The aim of this study was to investigate the association between the different components of impulsivity, assessed by neuropsychological tasks thought to tax different mechanisms of impulse control, and the 5-HTTLPR in a non-clinical sample.

## Methods

### Participants

We studied 127 self-assigned Caucasian-Brazilians, comprised of 86 undergraduate students and 41 graduates from two local universities communities, who were free of an Axis I diagnosis, as assessed by a psychiatrist using a structured interview (MINI-PLUS) and following DSM IV criteria. All participants were recruited through local advertisement at the universities.

The study was carried out in accordance with the Declaration of Helsinki. The Research Ethics Committee of the Universidade Federal de Minas Gerais approved all procedures, and subjects signed an informed consent after receiving a full explanation of the study. All subjects took part in this study on a volunteer basis, with no type of reward offered.

### Genotyping

Genotyping was performed as previously described (see in Corrêa et al. [19]); researchers involved in genotyping were blind to neuropsychological results.

### Neuropsychological Assessment

The neuropsychological assessment has been described elsewhere (see in Malloy-Diniz et al. [17]). Briefly, we used *Conner*'*s Continuous Performance Task* (CPT-II; omission and commission errors as measures of attentional and motor impulsivity) and the *Iowa Gambling Task* (IGT; the net score was used as a measure of non-planning, decision-making, -related impulsivity). Unlike CPT-II scores, high scores on the IGT indicate a low level of impulsivity. Two trained neuropsychologists – LFMD and SSCA administered the CPT-II and IGT.

### Analysis

Although there is no unanimity [20], some studies have assumed that the S allele is dominant and grouped the genotypes LS and SS [9], [11], [18], [19]. Using the same logic of these authors, we grouped S-carriers (LS + SS genotypes) and conducted comparisons of impulsivity scores of two-genotype (LL and LS + SS). Analyses were carried out separately by gender. Kolmogorov-Smirnov tests indicated that only omission scores of CPT-II were non-normally distributed for both genders. Thus, non-parametric tests were only performed for this measure related to attentional impulsivity. Parametric tests were performed for the scores of motor (commission errors of the CPT-II) and non planning (net score of the IGT) impulsivity. Comparisons of the distribution of genotypic frequencies were calculated using the chi-squared test. The significance level was 5% (*p*≤.05).

## Results

The overall average age of the sample was 29.5±11.8 years. Seventy five females ranging from to 18 to 57 years old (mean age 29±10.7) and fifty two males ranging from to 18 to 64 years old (mean age 30.2±13.2) participated in this study.

The genotypic frequencies of the female groups were 34.6% to the LL group, 51.4% to the LS group and 17.3% to the SS group. The genotype distribution was in Hardy-Weinberg equilibrium (x^2^ = 0.007; df = 1; *p* = 0.92). The number of participants into each genotype group is presented in Table 1.

**Table 1:** The number of female and male participants into each genotype group.

Gender	Genotype		
	LL	LS	SS
**Female**	26	36	13
**Male**	16	21	15

The genotypic frequencies of the male groups were 30.7% to the LL group, 40.3% to the LS group and 28.8% to the SS group. The genotype distribution was in Hardy-Weinberg equilibrium (x^2^ = 1.91; df = 1; *p* = 0.16). The number of participants into each genotype group is presented in Table 1.

**Figure 1:**
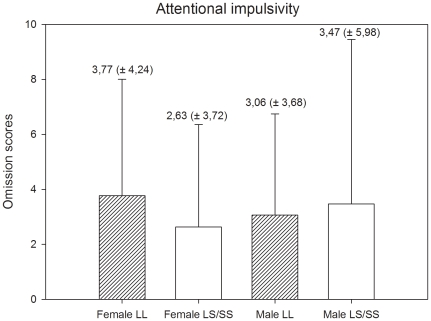
Attentional impulsivity scores and standard deviation of scores by gender and genotype.

**Figure 2:**
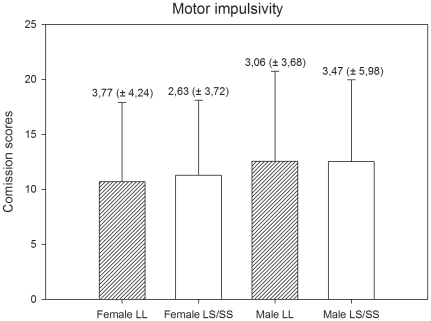
Motor impulsivity scores and standard deviation of scores by gender and genotype.

**Figure 3:**
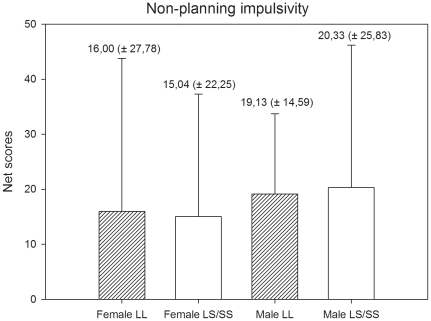
Non-planning impulsivity scores and standard deviation of scores by gender and genotype.

For both genders, we compared individuals with LL genotypes to individuals carrying an S-allele (LS + SS genotypes) using Student's *t-*tests and Mann-Whitney U tests. The analyses of females groups did not show differences in attentional (Z = 11.57, p = 0.11), motor (*t* (73) = 0.335, p = 0.72) and non-planning [*t* (73) = 0.63, p = 0.87) impulsivity. The same pattern of results was found in the analyses of male groups. No differences were found in attentional (Z = −0.316, p = 0.75), motor [*t* (50) = 0.01, p = 0.98) and non-planning [*t* (50) = −0.17, p = 0.86) impulsivity. The means and standard deviation of means of the impulsivity scores for females and males are presented in Figures 1, 2, and 3.

## Discussion

The present study is the first, to our knowledge, to use neuropsychological measures to investigate the association between the different facets of impulsivity and the 5-HTTLPR in a non-clinical sample. Corroborating the findings of Roiser et al. [7], our results did not show significant associations among impulsivities and genotypes (LL and LS plus SS).

The number of studies that did not find a significant relationship between 5-HTTLPR and motor impulsivity [7], [13], [14], [21] is greater than the number of studies that did find a significant association [11]. Discrepancy is also found in the analysis of attentional impulsivity. In that respect, our results are in agreement with Roiser et al. [7], who found no association between 5-HTTLPR genetic variation and attentional impulsivity, but contrast with the findings of Sakado et al. [14] who did find an association with this outcome.

Some methodological issues are relevant when discussing why some studies report significant relationship whereas others do not. Firstly, we can emphasize the question of how impulsivity is measured. A possible explanation for this discrepancy between results is that the self-report measures did not assess impulsivity in the same way as behavioral measures. Impulsivity is a complex paradigm; its definition and measurement are controversial. Therefore, the method of assessment of impulsivity greatly affects the experimental results. Laboratory tests present an advantage over questionnaires because questionnaires can introduce recall and interpretation biases. The use of neuropsychological computer measures has been recommended to determine impulsivity [12].

Other methodological issues are gender and race effects. There is some evidence that the effects of 5-HTTLPR genotype may depend on gender and race [14]. Studies have investigated Caucasian [7], [12] and Asian individuals [14], as well as specific gender [14] and mixed samples [7], [13], [19]. Research with similar methods need to be replicated to clarify specific relationships among 5-HTTLPR and impulsivities. It could provide a solid base of information to a future meta-analysis. As suggested by Umekage et al. [22], it might provide fruitful results by aiming at compensating a weakness of the studies, namely the reduced statistical power. A specific limitation of our study was the lack of inclusion of the triallelic method.

In conclusion, we did not find an association between impulsivity and the 5-HTTLPR. The allele L_G_ that has a transcriptional efficacy comparable to the S allele [23] was not investigated. Therefore, our next step is to investigate a possible association between impulsivity and the triallelic polymorphism in a larger sample.
